# What do Libyan doctors perceive as the benefits, ethical issues and influences of their interactions with pharmaceutical company representatives?

**DOI:** 10.11604/pamj.2013.14.132.2598

**Published:** 2013-04-06

**Authors:** Mustafa Ali Alssageer, Stefan Robert Kowalski

**Affiliations:** 1 Department of Pharmacology, Faculty of Medicine, Sebha University, Sebha, Libya; 2 School of Pharmacy and Medical Sciences, University of South Australia, Adelaide, Australia

**Keywords:** Pharmaceutical company interactions, drug information, physician perception, Pharmaceutical Company Representative

## Abstract

**Introduction:**

Evidence suggests that 80-90% of doctors in most countries across the world are frequently visited by pharmaceutical company representatives (PCRs). The objective of study to examine perceptions of Libyan doctors between August and October 2010, regarding the benefits, ethical issues and influences of their interactions with (PCRs).

**Methods:**

An anonymous questionnaire was circulated to 1,000 Libyan doctors in selected public and private practice settings in Tripoli, Benghazi and Sebha.

**Results:**

The major benefits of PCR visits reported in the 608 evaluable responses were; receiving new information about products (94.4%). The majority of doctors (75%) were not against the provision of gifts but were more comfortable if it was “cheap” (51%) and had educational value (51%). Doctors who received more printed materials, simple gifts or drug samples were less likely to disapprove of accepting gifts (p5]. Effective marketing can positively influence an individual's attitude towards a product and because there is an association between attitude, intention and behaviour [6], persuasive communication can generate a positive attitude and increase the potential for influence [7]. PCRs can accomplish behaviour change because they directly communicate with prescribers. During a visit they attempt to raise awareness of their products, provide product information and encourage a favourable attitude towards their company and product [8]. They employ verbal persuasion techniques and also provide other incentives such as gifts, free drug samples and sponsored educational events [2]. The provision of promotional gifts can be seen as a friendship building technique to reinforce the communication nexus between PCRs and doctors but it can also potentially erode professional barriers [9]. Contact between a PCR and a medical practitioner is therefore viewed by drug companies as a vital part of their marketing strategy and frequent visits, together with written promotional materials, gifts and other incentives, can help alter behaviour even if the initial attitudes towards a product were weak or unclear [10].

## Introduction

Pharmaceutical companies direct a major proportion of their promotional budgets towards the medical profession. In the United States of America (USA) in 2004 the amount allocated to the direct marketing of products to clinicians was $US7 billion [1]. Several factors influence physician drug prescribing but pharmaceutical company promotion has been reported as one of the primary influences on the drug selection process [2, 3]. The promotion and prescription of newer, more expensive drugs is also a major reason for rising prescription expenditure [4].

Pharmaceutical company representative (PCR) visits are one of the most effective and expensive tools used by companies to influence doctors’ prescription choices [5]. Effective marketing can positively influence an individual's attitude towards a product and because there is an association between attitude, intention and behaviour [6], persuasive communication can generate a positive attitude and increase the potential for influence [7]. PCRs can accomplish behaviour change because they directly communicate with prescribers. During a visit they attempt to raise awareness of their products, provide product information and encourage a favourable attitude towards their company and product [8]. They employ verbal persuasion techniques and also provide other incentives such as gifts, free drug samples and sponsored educational events [2]. The provision of promotional gifts can be seen as a friendship building technique to reinforce the communication nexus between PCRs and doctors but it can also potentially erode professional barriers [9]. Contact between a PCR and a medical practitioner is therefore viewed by drug companies as a vital part of their marketing strategy and frequent visits, together with written promotional materials, gifts and other incentives, can help alter behaviour even if the initial attitudes towards a product were weak or unclear [10].

Several studies have highlighted the considerable impact of PCR interactions on doctor's perceptions and their prescribing behaviour. For example as PCR visits increase, adherence to in-house prescribing guidelines decreases [11–13]. In a review of 16 studies, Wazana explained that interactions with pharmaceutical companies are associated with the following; negative impact on knowledge, incapacity to identify inaccurate or misleading claims about medication, positive attitudes to PCRs and their interactions, formulary addition requests for sponsored drugs that are not superior to existing formulary drugs, prescribing practices in favour of the promoted drug, rapid prescribing of new drugs and a decrease in the prescribing of generic drugs in favour of newer medications with no demonstrated advantages [2].

These impacts are one of the reasons that some countries and health institutes have instituted ethical codes and enacted regulations to minimize the negative influences of interactions with PCRs. Guidelines and regulations for drug promotion exist in many developed countries such as Canada and Australia, but do not exist or are outdated in many developing countries.

Libya has been privatising the pharmaceutical system since 2003, and the pharmaceutical industry is now allowed to market their products. Although PCRs are free to interact with health professionals, there are limited laws and regulations in Libya to standardize pharmaceutical promotional activities. The aim of this study was to examine the attitudes of Libyan doctors to the benefits, ethics and influences on prescribing practice from their interactions with PCRs.

## Methods

This publication examines responses from a study the first part of which has been previously been published in the Libyan Journal of Medicine [14]. A study employed a self-administered questionnaire that was circulated to 1000 Libyan physicians in Tripoli, Benghazi and Sebha. The study was conducted between August and October 2010. Inclusion criteria, questionnaire administration, and other methodological aspects of the study are detailed in a prior publication [14].

For this report, the questionnaire (Annex 1) sought the characteristics of the respondents and their practices, their frequency of involvement in pharmaceutical promotional activities and their attitudes towards the PCR interactions.

A Kruskal Wallis Test was employed to examine possible associations between the frequency of receiving promotional tools (printed materials, simple gifts and free samples) versus a doctor's attitude towards accepting gifts for three outcomes (No, Yes, and in some cases).

To further examine this relationship between a doctor's attitude toward receiving printed materials, simple gifts or drug samples, a logistic-regression analysis was performed. The population was divided into those practitioners who disapproved of accepting printed materials, simple gifts, drug samples versus those who fully approved, or approved in some cases. A cross tabulation analysis of doctors’ attitudes towards accepting gifts from PCRs was conducted to examine the impact of perceived educational value on gift acceptance, stratified by cost (expensive, medium or cheap). The study was ethics approved by the University of South Australia′s Human Research Ethics Committee.

## Results

Of the 1000 questionnaires circulated, 616 were collected. Eight questionnaires had incomplete data so were omitted from the final analysis. 608 (61%) of the returned questionnaires were therefore included for analysis. Demographic characteristics of study subjects have been detailed in previous published article [14]. Most respondents (n=423; 86%) reported that they had been given printed material (n=480; 79%), simple gifts (stationery, n=442; 73%) or drug samples (n=418; 69%) at least once during the last twelve months (Table 1).

**Table 1:** Frequency of receipt of printed materials, simple gifts and free samples versus response to question 5

Is it ethical to accept gifts from PCRs?					Logistic Regression# Analysis No vs. Yes or in some cases		
	No	Yes	In some cases	Total	sig	OR	95% CI
Printed materials*							
Never	47 (37)	33 (26)	48 (38)	128		1	
Once	29 (31)	22 (23)	43 (46)	94	0.393	1.281	0.72-2.25
2-5 times	52 (23)	52 (23)	119 (53)	223	0.008	1.897	1.18-3.05
>5 times	26 (16)	45 (28)	92 (56)	163	<0.001	3.057	1.76-5.31
Simple gifts*							
Never	43 (36)	37 (23)	74 (42)	154		1	
Once	59 (28)	38 (24)	69 (48)	166	0.156	1.411	0.87-2.27
2-5 times	42 (20)	55 (26)	112 (54)	209	0.001	2.226	1.39-3.54
>5 times	10 (13)	22 (28)	47 (59)	79	<0.001	3.841	1.84-8.01
Drug samples*							
Never	60 (32)	41 (22)	89 (47)	190		1	
Once	38 (26)	41 (28)	66 (46)	145	0.271	1.310	0.81-2.11
2-5 times	42 (22)	42 (22)	103 (55)	187	0.047	1.595	1.00-2.52
>5 times	14 (16)	28 (33)	44 (51)	86	0.008	2.392	1.25-4.57
Total	154	152	302	608			

# Significant (P < 0.05)

& “No” Responses versus (“yes” or “in some cases”) using “never accept” as the reference.

**Benefits from PCR interactions:** The three major perceived benefits reported by doctors from PCR visits were; receiving new information about products (n= 574; 94.4%), invitations to conferences (n=215; 35.4%) and receipt of gifts (n=132; 21.7%) (Table 2). A smaller proportion of respondents (n=28; 4.6%) reported the following other benefits; continuing medical education (n=2), free samples (n=9), meal invitations (n=2) or other non-specified benefits (n=15).

**Table 2:** Perceived benefits from interactions with pharmaceutical company representatives

	N	%
New information	574	94
Invitation to conferences	215	35
Gifts	132	22
Other*	28	5

* Medical Education (n=15), free samples (n=9), meal invitations (n=2) and others (n=15).

**Attitudes towards accepting PCR gifts:** Of the 608 respondents, a quarter of respondents (154; 25.3%) totally disapproved of accepting gifts from PCRs. This was balanced by an approximately equivalent number of respondents (n=152; 25%) who clearly approved. Approximately half the respondents (n=302; 49.7%) would accept gifts in some cases (Table 1).

**Frequency of receiving printed material, simple gifts and drug samples, and attitudes to accepting gifts:** A doctor's attitude towards the acceptance of gifts was significantly associated with the frequency they received printed materials, simple gifts and drug samples (pTable 1). Doctors who had received printed materials and simple gifts materials more than 5 times in the last year were more than three times as likely as those who never received materials to believe it is ethical to accept gifts from PCRs (unadjusted OR=3.05; pTable 1). Doctors who received free samples (on more than five occasions) were also more than twice as likely as those who had not received free samples to be agreeable to accepting gifts (unadjusted OR=2.3; p<0.01).

**Acceptance of gifts according to educational value and overall cost:** For respondents who did not disapprove of gift provision, just over half (n=262; 51%) reported that they would only accept educational gifts. By contrast (n=95; 18%) respondents reported that they would accept only non-educational gifts (n=11; 2.5%) (Table 3). Based only on a gift's cost, 51% of the recorded responses indicated cheap gifts were acceptable compared to 154 (30%) and 101(19%) of responses when the gifts were of medium or expensive value respectively. Over a half of the respondents’ responses (n=137; 52%) approved of cheap gifts but only if the gifts had educational value, compared to 37 (14%) of respondents’ responses who only approved of cheap non-educational gifts. For the expensive gift category, educational gifts were more likely to meet with approval (n=71, 70%) compared to expensive non-educational gifts (n=14, 14%). Of the 161 respondents’ responses that considered both educational and non-educational gifts to be ethically acceptable, there were fewer respondents (n=16; 10%) comfortable with accepting expensive gifts compared to a cheap gift (89; 55%).

**Table 3:** Analysis of the 518 respondents who did not disapprove of gifts further analysed based on educational content and cost of the gift

	Educational Value			
Cots of gift	Respondents who only approved of gifts with educational value	Respondents who only approved of non-educational gifts	Respondents who provided responses in both the educational and non-educational gifts categories*	Total
Cheap	137 (52)	37 (14)	89 (34)	263 (51)
Medium	54 (35)	44 (29)	56 (36)	154 (30)
Expensive	71 (70)	14 (14)	16 (16)	101 (19)
Total	262	95	161	518

**Figure 1:**
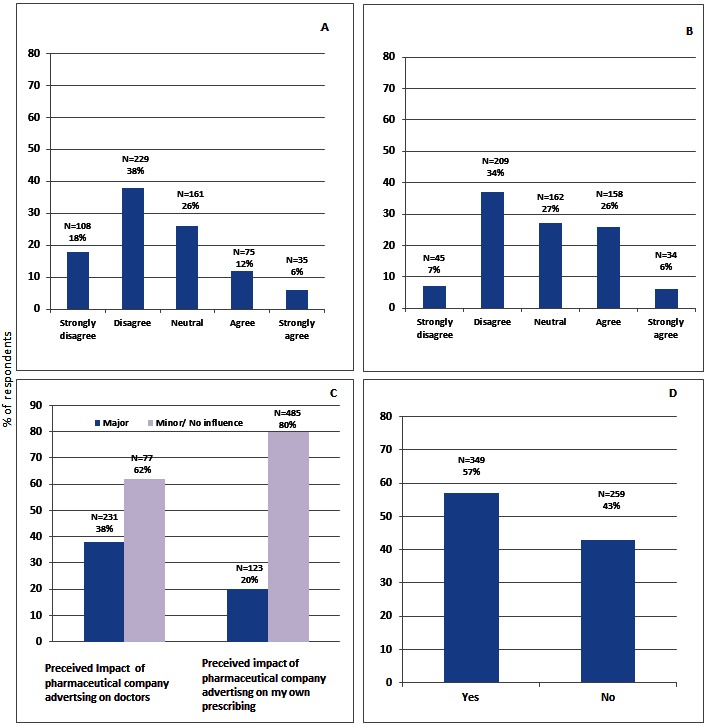
A: pharmaceutical company representatives information should be the main source of drug information that doctors receive?; B: Do Pharmaceutical Promotional activities decrease the likelihood of rational prescribing?; C: Impact of pharmaceutical promotion on prescribing; D: Is there a need to develop national polices to restrict PCRs interactions with doctors?

**PCRs and sources of drug information:** The majority of respondents (n= 337; 56%) disagreed or strongly disagreed with the statement that PCRs should be the main source of drug information. Nearly one fifth (n=110; 18%) of respondents however believed that PCRs should be the main source of drug information (Figure 1-A).

**Impact of pharmaceutical promotion on rational prescribing:** 254 (41.7%) respondents disagreed that pharmaceutical promotional activity decreased rational drug prescribing compared to 192 (32%) who agreed and 162 (26.7%) who reported a neutral response (Figure 1-B).

**Impact of pharmaceutical promotion on prescribing decisions:** The majority of respondents (377; 62%) reported that they believed pharmaceutical advertisements had minimal influence on doctors’ prescribing practices in general (Figure 1-C). When asked more specifically about their own prescribing, 485 (80%) respondents believed that promotional techniques had only a minor effect on their prescription decisions while 20% (n=123) believed that pharmaceutical advertisements had a major influence. Of the 231(38%) respondents who answered that pharmaceutical promotional activity had a major influence on doctors’ prescribing in general, 121 (52.4%) still believed that promotional activities had no influence or a minor influence only on their own prescription decisions (Figure 1-C).

**Developing policies for restricting doctor-PCR interactions:** Over a half of surveyed participants (n=349; 57%) reported that they approved of developing policies for restricting the interactions of PCRs with doctors (Figure 1-D).

**Doctor awareness of guidelines regarding PCR interactions:** Ninety-nine per cent of doctors (n=602/608,) had never read any guidelines regarding doctor-PCR interactions.

## Discussion

Pharmaceutical promotion has increased in Libya over the last decade however, there is little research analysing the impact of interactions with PCRs on the attitudes and prescribing behaviour of doctors in Libya. The results of this study provide some insights into doctors’ attitudes towards their interactions with PCRs.

Time, quality of, and accessibility of information are crucial determinants when addressing the usefulness of information sources [15]. Many studies [3, 16–18], including our research, have confirmed that doctors often use PCRs to update their drug knowledge. The majority of doctors (79%) received printed materials in the last year and 94% of the respondents surveyed reported that new information about pharmaceutical products was the main benefit they received from their interactions with PCRs. However, the majority of respondents (56%) disagreed that the information provided by PCRs should be their main source of drug information.

A United States (US) study reported that although physicians believe commercial sources were less accurate than non-commercial sources, they were still used more frequently [19]. In Thailand PCRs were also likely to be the initial source of information for most doctors, even though doctors considered their information to be less reliable [17]. Medical practitioners acquire their information by using resources that are easy to access, use, update, and that are flexible, free or low cost. Roughead et al. suggested that commercial information sources are often a more passive source of education and require less effort, are easily available and may be tailored to a doctor's request [8]. The provision of a brief verbal summary and edited printed material is an easier and more time efficient technique than reading and carefully appraising scientific journals.

The current study found a generally favourable perception toward PCR information. Although, as mentioned previously, the majority of respondents (n=337; 56%) disagreed that PCRs should be the main source of drug information, 18% (n=110) of respondents agreed/strongly agreed with the statement that PCR information should be the main source of drug information to doctors. In many developing countries, scientific sources of drug information are not easily available and the majority of medical professionals in developing countries have insufficient access to reliable information [20]. PCRs may be the only source of drug information available to some prescribers [21, 22]. The option to access other sources of prescribing information can provide both an alternative and/or a supplement to information provided by representatives. Published studies have reported that the quality of drug prescribing is increased if medical practitioners use independent sources of information and is decreased if the information used is provided by PCRs [23]. Unfortunately our questionnaire did not ask what other sources of drug information were available to Libyan prescribers.

In Libya, prohibiting or restricting PCR-doctor interactions may deprive some practitioners of their only source of new prescribing information and compromise health care. Instead of banning doctors’ interactions with PCRs, it is more important to create a variety of information sources that are quickly and easily accessible.

In other countries [3, 16, 24–26], prescriber perceptions of the value of the drug information provided included those clearly opposed, at least somewhat valuable, useful for new drugs, and important as an independent source of information. Gambrill and Bridges'Webb found that 56% of Australian doctors surveyed reported that they used PCRs as a regular source of information, but only 17% graded them as the most useful source used [27]. The provision of balanced, accurate and up to date company information during visits can be valuable, but may be accompanied by subtle or non-subtle promotional activities intended to induce behavioural change in a prescriber [8].

How therefore does the information provided by a PCR influence a doctor's prescribing? The majority (68%) of respondents in our study disagreed or were neutral about the statement that pharmaceutical promotion has a negative impact on rational drug prescribing. Sources of drug information affect a doctor's knowledge of therapy, influence attitude towards drug prescription and prescribing habits [28]. The lack of reliable independent drug information has been linked with irrational prescribing [29]. Evidence has demonstrated that doctors who rely more on commercial information prescribe more heavily, less rationally [5] and adopt new medicines more quickly [30, 31].

Although physicians may principally see PCR to receive information about new drugs, the visits allow PCRs the opportunity to build rapport and offer gifts. Approximately one third (n=215; 35.35%) of respondents indicated that the provision of gifts was one of the benefits of their interaction with PCRs. Most respondents (n=442; 73%) had received a simple gift at least once in the past 12 months. The majority of respondents (n=456; 75%) were not against the provision of gifts, but cheap gifts were more likely to be considered appropriate than middle value or expensive gifts. Only 19% (n=101) of respondents’ responses approved of expensive gifts and (n=71; 70%) of these responses considered that it only appropriate to accept an expensive gift if it had an educational purpose. By contrast only 14 respondents (14%) approved of non-educational expensive gifts. Consistent with a more favourable attitude to the receiving educational gifts, one fifth (n=132, 21.7%) of respondents indicated that receiving invitations to conferences was the one of benefits of their interaction with representatives and two respondents also reported continuing medical education as a benefit. Doctors were therefore more comfortable with accepting expensive gifts if they had educational value. The results of our study are also consistent with other studies of medical practitioner attitudes toward PCR gifts [32–34]. Expensive inducements or gifts are more easily accepted if they have an educational value.

Generally, doctors believe that regardless of education value, as the cost of a gift increased, so too did the potential of an ethical compromise [35]. This implies that they perceived an association between the value of a gift and its potential to influence prescribing decisions. McKinney examined the relationship between the cost of a gift and its potential to influence a physician′s prescribing decisions. They found that 15-24% of physicians believed that accepting a gift valued at $5 or less would influence a physician's prescribing decision, 40%-42% thought that there would be an influence if the gift was valued up to $50, and 80% agreed that a gift worth up to $1000 would compromise prescribing judgement [33]. To provide some direction, the Pharmaceutical Research and Manufacturers of America (PhRMA) Code (2002) [36] and The American Medical Association's (AMA) Council [37] guidelines of (1988) standardized permissible gifts to < $100 or as long as they benefit patients. Benefits received from PCR interactions and educational events do have potential patient benefits and it can be argued that small gifts do not significantly influence a prescriber's behaviour. However, the receipt of small low cost items can however still influence prescribing and these promotional tools have subtle influences on doctors’ decisions and are part of a social relationship building exercise by PCRs. In this study, doctors who received more printed materials, simple gifts and drug samples were less likely to disapprove of accepting gifts. This is consistent with other studies that have reported that prescriber perceptions about the appropriateness of gifts are influenced by their involvement in pharmaceutical promotional activities [2, 23, 38, 39].

Doctors may underestimate the impact of pharmaceutical promotion on their prescribing decisions. The majority of respondents (62%) in this study considered doctors’ prescribing practice to be either independent or minimally affected by pharmaceutical company promotions. This underestimation of the influence of pharmaceutical companies becomes far more significant when reporting about their own prescribing practices compared with those of their colleagues. Out of 608 surveyed prescribers only 20% acknowledged a major impact on their own prescribing while the majority (80%) reported that their own prescription choice was minimally or not at all influenced by pharmaceutical advertisements. The study found that the majority of doctors (n=121/231, 52.4%) who believed that pharmaceutical advertisements might have a major impact on their colleague's prescribing decisions, also denied such impact on their own medical judgement. This suggests that doctors feel they are less susceptible to the influence of promotions than their peers.

Lieb and Brandtonies also found that doctors believe their colleagues were three or four times more likely to be influenced than they were themselves. Only 6% admitted that they were often, or always influenced [40]. In the other words, physicians tend to deny or dismiss the influence of promotion on their prescribing [2]. This is consistent with research that indicates that individuals are prone to unintentionally optimistic biases in assessing themselves and attribute positive outcomes to themselves but negative outcomes to others [41]. Overconfidence or underestimating the negative impact of interaction of pharmaceutical company on doctors’ attitude is an important risk factor for being misled [42, 43]. Aldir et al. found that that in the USA 90% of medical practitioners and 87% of residents believed that they had adequate knowledge to critically understand information from commercial sources [35]. In many developing countries, a lack of awareness of evidence-based health care, guidelines for interaction with pharmaceutical companies, or long-term lack of access to unbiased information could result in undue influence from PCR pharmaceutical promotional techniques.

In developing countries there are usually limited legal statutes to direct appropriate promotional activities. In addition physicians receive little guidance on how to assess pharmaceutical promotional activities and understand the often subtle influence on their behaviour. The study found that almost all Libyan doctors (99%) were unaware of what constitutes appropriate PCR-medical practitioner interactions. A proportion of participants (43%) however also reported that they disapproved of developing policies that would restrict interactions with PCRs.

Healthy scepticism regarding any provided information can be a safeguard against inaccurate or misleading information planned primarily to influence those who are least sceptical [44, 45]. Burashnikova et al. compared answers to resident survey questions before and after an “anti-promotional” educational teaching course and found that post-survey there was a two-fold increase in the number of participants who considered pharmaceutical promotion to have a major influence on their colleagues’ prescribing practice, a near 3-fold decrease in the number of residents who considered respite gifts from pharmaceutical industry to be appropriate, and an increase (from 63.6% to 86.4%) in the number of respondents who disagreed with receiving inducements [38]. Similarly, Hopper et al found that after education about guideline on gifts to doctors from pharmaceutical companies, surveyed residents were more likely to agree that pharmaceutical gifts inappropriate [46]. Therefore, training to better understanding the marketing techniques used by the pharmaceutical industry, skills in literature and information assessment, as well as the principles of evidence-based medicine should be a fundamental part of undergraduate, postgraduate and continuing education.

The behaviour of individuals cannot be seen in isolation from the behaviour of others in the networks in which they operate. Since the behaviour of individuals is influenced by the behaviour of colleagues in social and practice settings, the use of restrictions or guidelines for interactions between health professionals and PCRs in medical institutes, can be important approaches to reduce the rate of interactions. An Australian study that examined a multi-doctor general practice clinic that instituted agreed guidelines for interactions with PCRs reported large reductions in the number of items of promotional materials (32%) and free samples (59%) received after policy adoption. In addition staff satisfaction with the agreed changes was high, and by not seeing PCRs during clinic time it was estimated that an extra 40 minutes per doctor was available for patient consultation [47].

Currently the website of the Libyan Board of Medical Specialities does not include any educational activities in relation to the rational use of medicines as advocated by the WHO [48]. Libyan doctors and PCRs should at least be aware of the general principles underlying product promotion. All promotional activities and their contents should be evaluated against the World Health Organization′s ethical criteria for medicinal drug promotion [49].

## Conclusion

The majority of surveyed doctors believed that their interactions with PCRs were beneficial since they received useful information regarding new drugs. They did not believe however that PCR's should be their main source of information. The receipt of PCR Incentives such as samples, gifts, and educational material can cultivate subconscious commercial or conflict of interest relationships with prescribers. Prescribers should have access to a variety of independent alternative sources of drug information so they can verify or refute information provided. Education regarding promotional techniques should also be provided in medical schools and be reinforced at an institutional level. Acceptance of personal vulnerability is a key of development own responsible behaviour management.

## Additional material

**Annex 1:** Study questionnaire

## References

[1] Kaiser Family Foundation Prescription Drug Trends May 2010 Update. http://www.kff.org/rxdrugs/upload/3057_07.pdf.

[2] Wazana A (2000). Physicians and the Pharmaceutical Industry - Is a Gift Ever Just a Gift?. JAMA..

[3] Kisa S (2006). Factors that Influence Prescribing Decisions among Turkish Physicians. Clinical Research & Regulatory Affairs..

[4] Moynihan R (2003). Who pays for the pizza? Redefining the relationships between doctors and drug companies. 1: entanglement. BMJ..

[5] Caudill TS, Johnson MS, Rich EC, McKinney WP (1996). Physicians, pharmaceutical sales representatives, and the cost of prescribing. Arch Fam Med..

[6] Bohner G, Wanke M (2002). Attitudes and attitude change.

[7] Cialdini RB, Petty RE, Cacioppo JT (1981). Attitude and attitude change. Annual Review of Psychology..

[8] Roughead L (1996). The pharmaceutical representative and medical practitioner encounter: Implications for quality use of medicines (MSc thesis).

[9] Katz D, Caplan AL, Merz JF (2003). All gifts large and small: toward an understanding of the ethics of pharmaceutical industry gift-giving. Am J Bioeth..

[10] Bem DJ (1967). Self-Perception: An Alternative Interpretation of Cognitive Dissonance Phenomena. Psychol Rev..

[11] Muijrers PE, Grol RP, Sijbrandij J, Janknegt R, Knottnerus JA (2005). Differences in prescribing between GPs. Impact of the cooperation with pharmacists and impact of visits from pharmaceutical industry representatives. Fam Pract..

[12] Carlzon D, Gustafsson L, Eriksson A, Rigner K, Sundstrom A, Wallerstedt S (2010). Characteristics of primary health care units with focus on drug information from the pharmaceutical industry and adherence to prescribing objectives: a cross-sectional study. BMC Clinical Pharmacology..

[13] van Duijn HJ, Kuyvenhoven MM, Schellevis FG, Verheij TJ (2005). Determinants of prescribing of second-choice antibiotics for upper and lower respiratory tract episodes in Dutch general practice. J Antimicrob Chemother..

[14] Alssageer MA, Kowalski SR (2012). A survey of pharmaceutical company representative interactions with doctors in Libya. Libyan J Med.

[15] Leckie GJ, Pettigrew KE, Sylvain C (1996). Modeling the Information Seeking of Professionals: A General Model Derived from Research on Engineers, Health Care Professionals, and Lawyers. The Library Quarterly.

[16] Ben Abdelaziz A, Harrabi I, Rahmani S, Ghedira A, Gaha K, Ghannem H (2003). Attitudes of general practitioners to pharmaceutical sales representatives in Sousse. East Mediterr Health J..

[17] Layton MR, Sritanyarat W, Chadbunchachai S, Wertheimer AI (2007). Sources of information for new drugs among physicians in Thailand. Pharm World Sci..

[18] Prosser H, Almond S, Walley T (2003). Influences on GPs’ decision to prescribe new drugs-the importance of who says what. Fam Pract..

[19] McCue JD, Hansen CJ, Gal P (1986). Physicians’ opinions of the accuracy, accessibility, and frequency of use of ten sources of new drug information. South Med J..

[20] Kale R (1994). Health information for the developing world. BMJ..

[21] Lexchin J, Kaur SR (1995). Deception by design: pharmaceutical promotion in the third world.

[22] Jafar TH, Jessani S, Jafary FH, Ishaq M, Orakzai R, Orakzai S (2005). General practitioners’ approach to hypertension in urban Pakistan: disturbing trends in practice. Circulation..

[23] Caamaño F, Figueiras A, Gestal-Otero JJ (2002). Influence of commercial information on prescription quantity in primary care. Eur J Public Health..

[24] McGettigan P, Golden J, Fryer J, Chan R, Feely J (2001). Prescribers prefer people: The sources of information used by doctors for prescribing suggest that the medium is more important than the message. Br J Clin Pharmacol..

[25] Oshikoya KA, Oreagba I, Adeyemi O (2011). Sources of drug information and their influence on the prescribing behaviour of doctors in a teaching hospital in Ibadan, Nigeria. Pan Afr Med J..

[26] Magzoub MA, Neyaz Y, Khoja T, Qureshi NA, Haycox A, Walley T (2011). Determinants of physicians’ medication prescribing behaviour in primary care in Riyadh City, Saudi Arabia. East Mediterr Health J..

[27] Gambrill J, Bridges-Webb C (1980). Use of sources of therapeutic and prescribing information by general practitioners. Aust Fam Physician..

[28] Figueiras A, Caamaño F, Gestal-Otero JJ (2000). Influence of physician's education, drug information and medical-care settings on the quality of drugs prescribed. Eur J Clin Pharmacol..

[29] Lexchin J (1990). Prescribing by canadian general practitioners: review of the english language literature. Can Fam Physician..

[30] Mapes R (1977). Aspects of British general practitioners’ prescribing. Med Care..

[31] Strickland-Hodge B, Jepson MH (1982). Identification and characterization of early and late prescribers in general practice. J R Soc Med..

[32] Steinman MA, Shlipak MG, McPhee SJ (2001). Of principles and pens: attitudes and practices of medicine housestaff toward pharmaceutical industry promotions. Am J Med..

[33] McKinney WP, Schiedermayer DL, Lurie N, Simpson DE, Goodman JL, Rich EC (1990). Attitudes of internal medicine faculty and residents toward professional interaction with pharmaceutical sales representatives. JAMA..

[34] Thomson AN, Craig BJ, Barham PM (1994). Attitudes of general practitioners in New Zealand to pharmaceutical representatives. Br J Gen Pract..

[35] Aldir RE, Jarjoura D, Phinney M, Poordad F, Gutierrez R, Marnejon T (1996). Practicing and resident physicians’ views on pharmaceutical companies. J Contin Educ Health Prof..

[36] (2002). Pharmaceutical Research and Manufacturers of America PhRMA code on interactions with healthcare professionals. http://www.phrma.org/files/phrma%20code.pdf.

[37] American Medical Association (1990). Ethical Guidelines for Gifts to Physicians from Industry. http://www.ama-assn.org/ama/pub/physician-resources/medical-ethics/code-medical-ethics/opinion8061.page.

[38] Burashnikova IS, Ziganshin AU, Ziganshina LE (2008). Attitudes to pharmaceutical promotion techniques among healthcare professionals in the Republic of Tatarstan, Russia. International Journal of Risk & Safety in Medicine..

[39] Haayer F (1982). Rational Prescribing and Sources of Information. Social Science & Medicine..

[40] Lieb K, Brandtönies S (2010). A survey of german physicians in private practice about contacts with pharmaceutical sales representatives. Dtsch Arztebl Int..

[41] Dana J, Loewenstein G (2003). A social science perspective on gifts to physicians from industry. JAMA..

[42] Sagarin BJ, Cialdini RB, Rice WE, Serna SB (2002). Dispelling the illusion of invulnerability: the motivations and mechanisms of resistance to persuasion. J Pers Soc Psychol..

[43] Weinstein ND, Lyon JE (1999). Mindset, optimistic bias about personal risk and health-protective behaviour. British Journal of Health Psychology..

[44] Friestad M, Wright P (1994). The Persuasion Knowledge Model: How People Cope with Persuasion Attempts. Journal of Consumer Research.

[45] Giarlo M (2006). The role of skepticism in human-information behavior: a cognitive-affective analysis. Library Student Journal.

[46] Hopper JA, Speece MW, Musial JL (1997). Effects of an educational intervention on residents’ knowledge and attitudes toward interactions with pharmaceutical representatives. J Gen Intern Med..

[47] Spurling G, Mansfield P (2007). General practitioners and pharmaceutical sales representatives: quality improvement research. Qual Saf Health Care..

[48] Mustafa AA, Kowalski S (2010). A need for the standardization of the pharmaceutical sector in Libya. Libyan J Med..

[49] World Health Organization Ethical criteria for medical drug promotion. http://www.who.int/medicinedocs.

